# Reconstruction or replacement? A challenging question in surgical treatment of complex humeral head fractures in the elderly

**DOI:** 10.1007/s00402-021-04124-3

**Published:** 2021-08-25

**Authors:** M. Müller, F. Greve, M. Crönlein, M. Zyskowski, S. Pesch, P. Biberthaler, C. Kirchhoff, M. Beirer

**Affiliations:** Department of Trauma Surgery, Klinikum rechts der Isar, Technical University Munich, Ismaningerstraße 22, 81675 München, Germany

**Keywords:** Proximal humeral fracture, Locking plate fixation, Reversed total shoulder arthroplasty, Complication, Revision, Outcome

## Abstract

**Introduction:**

Surgical treatment of complex humeral head fractures in the elderly is challenging due to osteoporotic bone, comorbidities and reduced compliance. The treatment strategy (reconstruction versus replacement) should allow for a functional aftercare and result in a high patient satisfaction. Major complications leading to surgical revision are crucial and should be avoided. The purpose of this study was to analyse the major complication rate leading to surgical revision and the patient-based outcome in complex humeral head fractures of the elderly population treated either using locking plate fixation (LCP) or reversed total shoulder arthroplasty (rTSA).

**Materials and Methods:**

All patients older than 65 years surgically treated due to a four-part fracture of the proximal humerus between 2003 and 2015 were enrolled in our retrospective study. Major complications and revision rates were recorded and functional outcome was assessed using the Munich Shoulder Questionnaire (MSQ) allowing for qualitative self-assessment of the Shoulder Pain and Disability Index (SPADI), of the Disability of the Arm, Shoulder and Hand (DASH) score and of the Constant Score.

**Results:**

A cohort of 103 patients with a mean age of 73.4 ± 6.2 years suffering from four-part fractures of the humeral head were enrolled. 63 patients were treated using the LCP fixation compared to 40 rTSAs. There were no significant differences in the patient-reported functional outcome. The revision rate was significantly higher in the LCP group (10/63; 15.9%) compared to the rTSA group (1/40; 2.5%). Reasons for revision were avascular head necrosis, cut-out of screws, secondary dislocation of the greater tuberosity and hypersensitivity to metal.

**Conclusions:**

Reversed total shoulder arthroplasty and locking plate fixation are both established surgical procedures for the management of complex proximal humerus fractures in the elderly leading to similar functional results. However the revision rate in the rTSA group was significantly lower. Primary rTSA should, therefore, be favoured in multimorbid elderly patients with an increased complication risk to avoid repeated anaesthesia.

## Introduction

In general proximal humerus fractures (PHFs) occur most commonly in elderly patients with an average age of 66 years whereas the majority of patients is female [1]. Along with fractures of the proximal femur and distal radius, PHF presents the third most common fragility fracture, often associated with osteoporosis [2]. Low bone mass, deficits in bone geometry and microarchitecture in combination with a higher risk of falling lead to a higher incidence of complex fracture patterns in terms of three- and fourt-part fractures according to Neer [1, 3, 4] in this patient cohort. Surgical treatment of these complex fractures in general is challenging, especially in older patients with comorbidities often resulting in a continuous modification and development of current surgical techniques.

The advent of the locking plate technology was considered as a breakthrough for the treatment of PHF in the elderly [5]. Advantages of this locking plate technology such as reduced friction and polyaxial locking screw positioning were promising, thus leading to a significantly increased use. Unfortunately, complication rates including surgeon-related problems (e.g. intraarticular screw positioning, screw loosening) as well as loss of reduction, screw cut-out, head necrosis and nonunion were considerably high [6–8]. The overall complication rate in patients > 60 years was reported as high as up to 45% leading to revision surgery in 18% of the patients within 1 year [7].

However, revision surgery along with repeated general anaesthetical procedures is crucial especially in the elderly patients due to a possible occurrence of postoperative cognitive dysfunction (POCD = the patient does not return to his baseline cognition function) [9]. Furthermore, cognitive impairment in the elderly can lead to a decline in function, loss of personality and relationships as well as enormous community, social and healthcare costs [10].

In addition, secondary conversion to rTSA as salvage procedure for failure of LCP fixation in PHF in the elderly leads to significantly worse results compared to primary rTSA [11, 12]. Every effort should be made to avoid complications leading to revision in this fragile patient collective. To be able to better decide which surgical procedure should be preferred it is important to understand the clinical outcome following either LCP fixation or primary rTSA.

Therefore, the purpose of this study was to assess the patient-based outcome as well as the major complications leading to surgical revision following primary LCP and rTSA, respectively, in treatment of four-part PHF in the elderly. We suppose that with the increasing experience in the field of shoulder arthroplasty, this could be the safer type of treatment of complex humeral head fractures in geriatric patients.

## Materials and methods

### Patients

The study protocol was approved by the local ethics committee. For this retrospective study the in-house fracture register was searched for patients older than 65 years suffering from four-part fractures of the proximal humerus surgically treated at our level I trauma center. Preoperative standard radiographs of the shoulder (anterior–posterior view and Y-view orthogonal to the anterior–posterior view) and additional computed tomography (CT) according to the fracture pattern were evaluated. Injuries were considered four-part fractures if the fracture separated the greater tuberosity, lesser tuberosity, humeral head and humeral shaft. This is in accordance with the four-segment approach of the Neer classification [13], although the original criteria (dislocation > 1 cm, tilt > 45°) were not necessarily fulfilled. Multiple trauma patients and patients with injuries of the same arm as the proximal humerus fracture were excluded. Written informed consent was obtained from each patient.

### Surgical technique and rehabilitation

In all enrolled patients surgery was performed using a deltopectoral approach in a beach chair position with the affected arm in a mobile position.

#### Group I—LCP

At first for performing locking plate fixation the separated tuberosities were fixed with tension band sutures to manipulate and reduce the free tuberosity fragments. Subsequently the humeral head was reduced and in case of medial metaphyseal fragmentation, the medial column was restored to avoid varus collapse. If necessary, the fracture reduction was temporarily secured using K-wires. Finally, the plate was fixed to the humeral shaft and head and the tuberosity sutures were passed through the small holes of the plate to fix the tuberosities.

#### Group II—rTSA

For the performance of a reversed total shoulder arthroplasty the separated tuberosities were fixed with tension band sutures for later fixation to the humeral shaft. The humeral head and all loose bone fragments were removed. The glenoid was exposed and prepared for implantation of the baseplate and the glenosphere. The humeral shaft was then exposed, prepared and the humeral trial prosthesis was placed. In the next step the prosthesis was reduced with different inlays to confirm proper joint tension and stability. The intramedullary cavity was prepared and cemented fixation of the definitive prosthesis was performed. After hardening of the cement the prosthesis was reduced and achievement of correct soft tissue tension was tested. If present the tuberosities were fixed to the stem to improve stability and function of the arthroplasty.

Postoperatively the arm was immobilized in a sling for 6 weeks. The patients were allowed to start physiotherapy on the first postoperative day following a standard rehabilitation protocol: active-assisted abduction and flexion were restricted to 90° for the first six weeks. Weight-bearing was not allowed during this period. With decreasing pain, this training was extended and movements across the horizontal plane were allowed.

### Follow-up

Follow-up was performed using a patient reported outcome measurement (PROM) tool, the so-called Munich Shoulder Questionnaire (MSQ) [14]. For patient-based outcome assessment the Munich Shoulder Questionnaire (MSQ) was sent to the patients by mail. This patient-reported outcome questionnaire presents a universally applicable instrument for the self-assessment of the shoulder function. It was developed for an effective follow-up of shoulder patients allowing for a quantitative assessment of the Shoulder Pain and Disability Index (SPADI), the Disability of the Arm, Shoulder and Hand (DASH) score and the Constant Score even without the examination by a clinician. The MSQ has been validated previously and its accuracy and effectiveness for follow-up evaluation was demonstrated [15, 16].

Major complications leading to surgical revision were assessed reviewing the electronic medical record of each patient. Radiographs of all patients who underwent revision surgery were analyzed regarding individual reasons for revision.

### Statistics

Data were given as arithmetic mean ± standard deviation. The results were compared using the Mann–Whitney *U *test for continuous variables or Chi-Quadrat-test for dichotome variables. Further a logistic regression analysis was performed to control for potential cofounders. A *p *value < 0.05 determined statistical significance. Statistics were calculated using commercially available programs (IBM SPSS Statistics for Windows, Version 22; Armonk, NY, USA).

## Results

The in-house fracture register revealed 375 patients suffering from four-part fractures of the proximal humerus between August 2007 and September 2015. These patients were contacted by mail. 103 patients answered in time and were consecutively enrolled. 63 patients (46 females, 17 males) were allocated to the LCP group (group I) and 40 patients (34 females, 6 males; n.s.) to the rTSA group (group II). Mean follow-up accounted for 52.5 ± 36.2 months in group I and for 33.5 ± 17.6 months in group II (*p* < 0.05). The LCP-group had a mean age of 71.4 ± 4.8 years compared to group II with 76.8 ± 6.8 years (*p* < 0.05). Time to surgery was significantly shorter in the locking plate fixation group (group I 2.3 ± 2.2 days; group II 4.3 ± 3.9 days; *p* < 0.05).

**Fig. 1:**
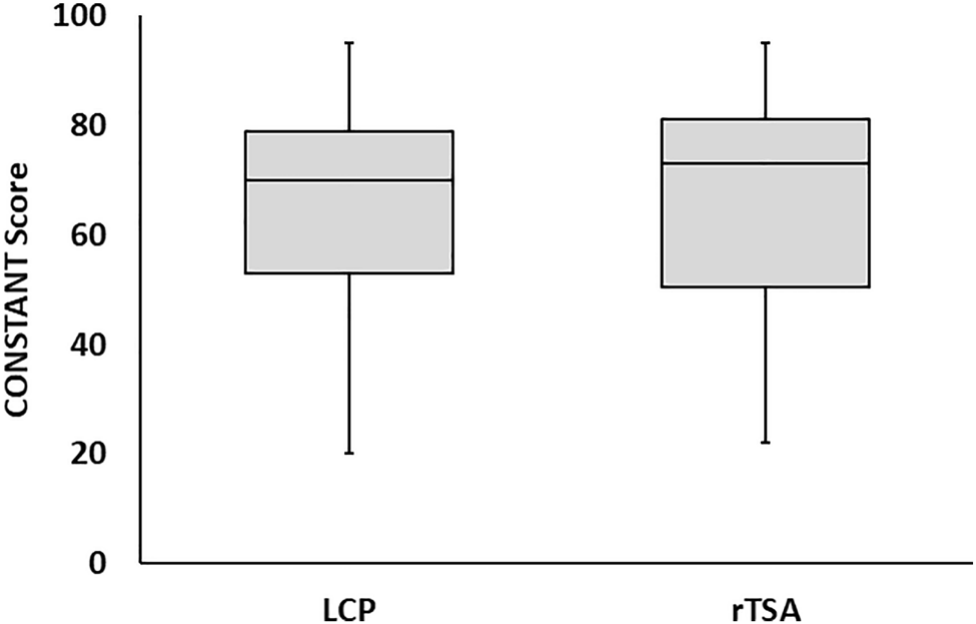
Boxplot diagraphs comparing the results of the CONSTANT-Score compared between the group with plate osteosynthesis (ORIF) and reversed shoulder arthroplasty (rTSA)

Regarding the patient-based outcome assessment no significant differences between group I and group II were found (mean MSQ 77.3 ± 18.0 vs. 72.2 ± 27.1, n.s.; mean SPADI 80.4 ± 20.6 vs. 75.1 ± 28.9, n.s.; mean DASH score 18.4 ± 20.2 vs. 19.6 ± 24.1, n.s.; mean Constant Score 66.6 ± 17.1 vs. 64.8 ± 24.2 points, n.s.) (Fig. 1). Male and female patients did not show significantly different MSQ results (74.6 ± 21.8 vs. 75.6 ± 22.2 n.s.)

**Fig. 2:**
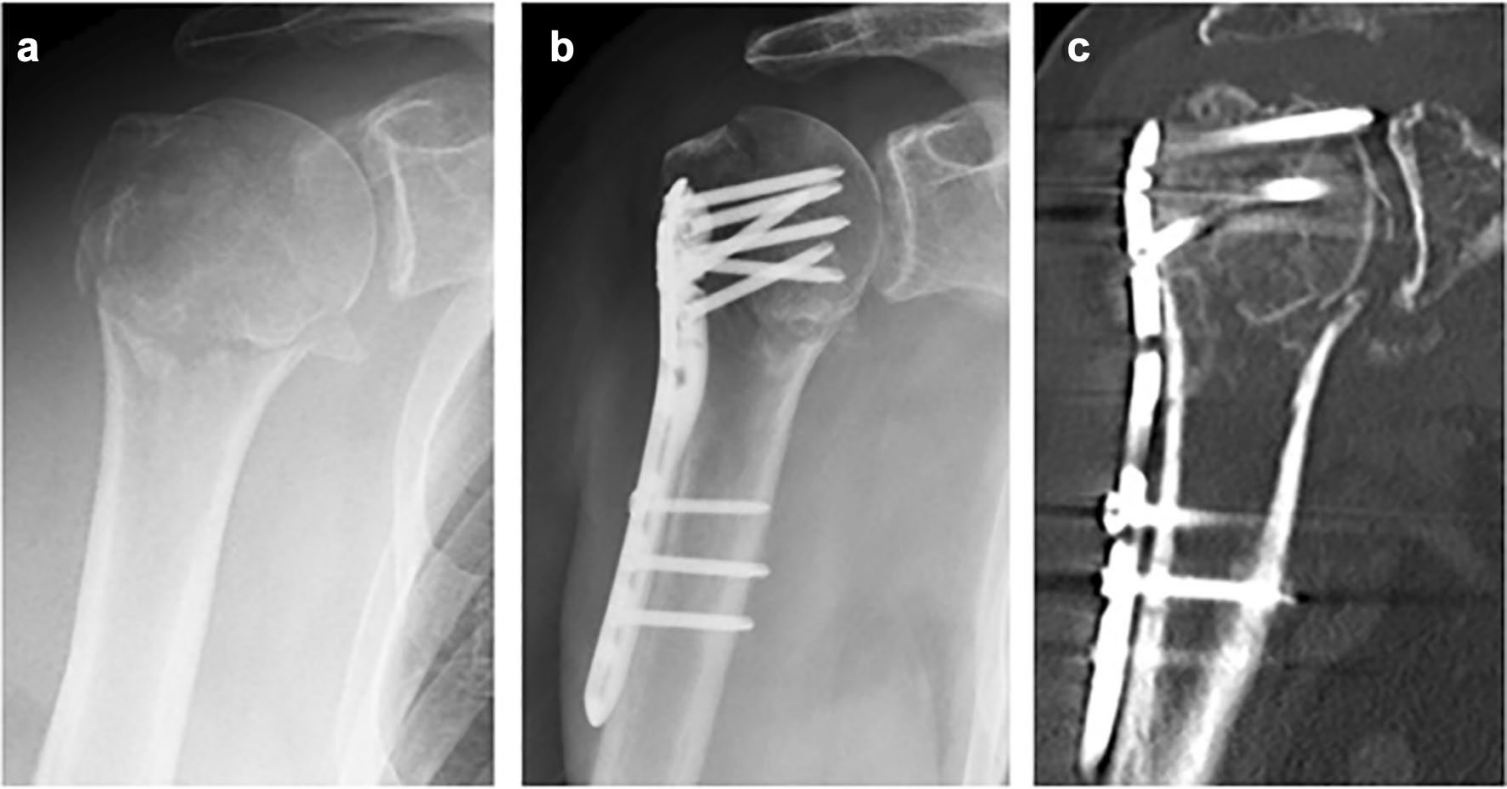
Case presentation. A 74 year old woman presented with a four-part fracture of the right humeral head. Open reduction and internal fixation was performed 1 day after the trauma. **a** preoperative ap-view of the fracture; **b** postoperative ap-view after reconstruction using a locking plate osteosynthesis; **c** CT scan two months postoperative shows a collapse of the humeral head with secondary dislocation of the screws

The revision rate was significantly higher in the LCP group (10/63 = 15.9%) compared to the rTSA group (1/40 = 2.5%; *p* = 0.032) (Table 1). A multivariate analysis controlling for the confounding variables age and gender was performed (Table 2) and confirmed the operation type as an independent risk factor for revision surgery (*p* = 0.049). Reasons for revision in group I were avascular humeral head necrosis (*n* = 3), secondary dislocation of tuberosity fragments (*n* = 3), cut-out of screws (*n* = 2) and postoperative haematoma (*n* = 2). Table 3 presents an overview of all patients who developed postoperative complications leading to revision surgery. Radiographs of a patient developing avascular head necrosis are shown in Fig. 2.

**Table 1:** Functional scores and revision rate for all 103 study patients

	All patients	Group I	Group II	*p*
*n*	103	63	40	
MSQ score	75.3 ± 22.0	77.3 ± 18.0	72.2 ± 27.1	0.758
SPADI score	78.3 ± 24.2	80.4 ± 20.6	75.1 ± 28.9	0.277
DASH score	18.9 ± 21.7	18.4 ± 20.2	19.6 ± 24.1	0.776
Constant score	65.9 ± 20.1	66.6 ± 17.1	64.8 ± 24.2	0.665
Revision	11/103 (10.7%)	10/63 (15.9%)	1/40 (2.5%)	0.032*

Data presented as mean ± SD or *n* (%)

*Statistically significant

**Table 2:** Logistic regression analysis of independent risk factors for revision operation

	OR	95% CI	*p *value
Age	1.049	0.925–1.189	0.455
Gender (male)	0.889	0.207–3.828	0.875
Operation type (ORIF)	9.673	1.003–93.09	0.049*

*OR *odds ratio, *CI *confidence interval

*Statistically significant

**Table 3:** List of ten cases with reported complications leading to surgical revision

Patient N^o^	Age/gender	Time to operation (days)	Hertel criteria	Treatment	Complication
1	68 years, female	1	HD ( +), SC ( +), AN (−)	ORIF	AVN,
2	77 years, female	2	HD ( +), SC (−), AN (−)	ORIF	Cut-out
3	74 years, female	1	HD ( +), SC (−), AN (−)	ORIF	AVN
4	74 years, ma le	1	HD (−), SC (−), AN (−)	ORIF	Postoperative hematoma
5	79 years, female	1	HD ( +), SC (−), AN (−)	ORIF ORIF	Cut-out
6	74 years, femearsale	0	HD ( +), SC (−), AN (−)	ORIF	Postoperative hematoma
7	77 years, female	3	HD ( +), SC ( +), AN (−)	rTSA	Metal hypersensitivity
8	70 years, female	0	HD ( +), SC ( +), AN (−)	ORIF	Secondary dislocation of GT
9	69 years, male	4	HD ( +), SC ( +), AN (−)	ORIF	Secondary dislocation of GT
10	78 years, male	5	HD (−), SC ( +), AN (−)	ORIF	Secondary dislocation of GT
11	76 years, female	2	HD ( +), SC ( +), AN (−)	ORIF	AVN

*HD *hinge displacement, *SC *short calcar segment (< 8 mm), *AN *anatomical neck fracture, *AVN *avascular head necrosis, *n.a. *not available, *GT *greater tuberosity

In group II one patient presented with pain, reddening and overheating of the concerned shoulder 11 months postoperatively. Due to clinical suspicion of a low-grade infection complete removal of the arthroplasty was performed. However, incubation of tissue samples and the polyethylene inlay after sonification showed no bacterial growth. Despite a missing history of metal allergy through contact jewelry or clothing accessories a skin patch test was performed at the department of dermatology confirming a metal hypersensitivity towards cobalt and nickel. Thus a hypoallergenic rTSA was implanted and the patient reported no further complaints.

## Discussion

Surgical treatment of proximal humeral fractures in the elderly is a challenging task due to osteoporotic bone, comorbidities and reduced compliance in this patient cohort. While in theory the locking plate technology presents clear advantages, the failure rate in PHFs of geriatric patients is quite high with a reoperation rate of 18% in four-part fractures. Conversion to rTSA after failure of locking plate fixation leads to functional improvement and pain relief, but functional scores decrease consequently and the complication rate is higher compared to primary rTSA in fracture treatment [12]. Therefore, the purpose of this study was to assess the patient-based outcome and the failure rate leading to surgical revision of LCP and primary rTSA in four-part PHFs of the elderly population.

The Munich Shoulder Questionnaire was used to assess the functional outcome and patients’ postoperative satisfaction. It allows for a self-assessment of the SPADI, the DASH and the Constant Score representing the major scores for the assessment of shoulder function and focuses on the subjective patient satisfaction which does not necessarily correlate with the physician-based examination [17]. The functional outcome with a mean Constant Score of 66.6 ± 17.1 points in group I and 64.8 ± 24.2 points in group II did not show significant differences and is comparable to the results of other authors [18–20]. Giardella et al. [18] also compared the outcome of ORIF versus rTSA of three- and four-part fractures in elderly patients and reported a better functional outcome for the arthroplasty group assessed with the constant score. Prima facie, the mean Constant Score of the presented collective with about only two-thirds of the highest Constant Score of 100 seems to be poor. However, the total Constant Score decreases with age for both genders [21]. According to Tavakkolizadeh et al. [21] a Constant Score between 78 and 80 points is equivalent to a normal shoulder function in patients over the age of 70. Taking this into account, the results of the presented study can be rated as good age-adjusted Constant Score results corresponding to a good recovery of the injured and surgically treated shoulder.

Avascular head necrosis (AVN), postoperative loss of reduction, screw cut-out and non-union are common major complications in humeral head-preserving surgical techniques often leading to surgical revision. In the presented study AVN appeared in three patients in group I. To estimate the risk of this crucial complication, Hertel et al. defined the length of the dorsomedial metaphyseal extension, the integrity of the medial hinge and some specific fracture types as most relevant factors for developing ischemia of the humeral head [22]. In the presented three cases with AVN the length of the medial metaphyseal head extension was very short, in two of three cases the medial hinge was displaced (see Table 3). Thus according to Hertel et al., the head perfusion was at risk. However, locking plate fixation was performed since further ischemia criteria were missing. In addition, the factors defined by Hertel can only be considered as recommendations because the achievement of adequate reduction and stable conditions even in initially ischemic humeral heads can lead to revascularization [23].

Deterioration of bone quality in geriatric patients with additional weakening due to further comorbidities (such as osteoporosis) and influence of medication decreases the resistance of bone against repeated load [24]. Hence premature failure of the bone around screws following fracture fixation can lead to cut-out of metal hardware [25] observed twice in group I. Bone cement augmentation to prevent cut-out of implants in geriatric patients suffering from proximal femur fractures was successfully performed even in revision surgery [26]. Thus cement augmentation also of humeral head screws was developed and biomechanical investigations showed an increased primary stability. In a clinical study, Katthagen et al. showed similar functional outcomes but significantly less screw cut-out in cement augmentation of locking plate fixation of PHF in the elderly compared to locking plate fixation only [27].

Fixation of small tuberosity fragments can be achieved by rotator cuff sutures placed into the subscapularis tendon, the supraspinatus and the infraspinatus tendon just superficial to the tendon’s bony insertion [28]. However, we observed secondary dorsocranial dislocation of initial anatomically reduced greater tuberosity fragments in two cases in the early postoperative period. In these patients the suture fixation of the infraspinatus tendon was not performed resulting in lower stability of the greater tuberosity.

In recent decades, metal hypersensitivity (MHS) as reason for implant failure gained more and more attention in orthopedic surgery. In the general population the frequency of cutaneous allergies with immunological reactions especially to nickel, cobalt and chromium is estimated of up to 13% [29]. Patients suffering from MHS following total hip and total knee arthroplasty present with periprosthetic joint pain, effusions and cutaneous eczematous rash [30]. In our study one patient reported periprosthetic joint pain and skin redness 11 months after implantation of a reversed total shoulder arthroplasty. Reasons for joint replacement failure such as infection or mechanical issues of size, placement or orientation were excluded. The patient reported no history of metal allergy; however, skin patch testing following removal of the prosthesis confirmed MHS against nickel and cobalt. Thus re-implantation of a hypoallergenic rTSA was performed and the patient reported no further complaints. Nevertheless, the clinical relevance of MHS remains unclear to the orthopedic surgeon. Especially in the acute fracture situation, comprehensive preoperative screening via skin patch testing would delay surgical treatment. Depending on local facilities further skin testing of patients with a positive metal allergy history (such as contact jewelry or clothing accessories [30]) constitutes an individual decision.

In the presented study the mean follow-up was significantly shorter in group II (33.5 ± 17.6 months) compared to group I (52.5 ± 36.2 months; *p* < 0.05). The shorter follow-up in group II might be due to the lower incidence of rTSA in relation to LCP fixation at the beginning of the study in 2007. During the study period rTSA was increasingly performed due to good postoperative results [31]. Data from the Finnish Arthroplasty Register and the Finnish National Hospital Discharge Register during 2004–2015 confirm this trend: the total number of reverse shoulder arthroplasty was rapidly increasing by 4500%, respectively [32].

In a systematic review the reoperation rate following open reduction and internal fixation (ORIF) of PHFs was significantly higher compared to rTSA after a similar follow-up of 30–34 months, supporting our findings [33]. Nevertheless, the shorter follow-up of the rTSA group in our study collective could be one factor for the lower revision rate in group II. One can expect that the low revision rate of 2.5% in the rTSA group will increase over time. Van Ochten et al. reported long-term results of reversed shoulder arthroplasty with a revision rate of 8.2% after 9 years [34]. Although this is a substantial increase, the long-term revision rate of plate fixation is still considerably higher [35].

The evaluation objective of the functional outcome, of the complication and revision rate following LCP and rTSA in the elderly is to facilitate the decision which procedure should be preferred in daily clinical practice. Spross et al. developed a therapeutic algorithm based on the activity level and the general health status. According to this algorithm rTSA is recommended for patients older than 70 years with four-part fractures without valgus impaction or patients older than 70 years with valgus impacted four-part fractures and tuberosity displacement > 1 cm towards the humeral head. Despite a high revision rate in the ORIF group the authors observed very satisfying functional outcomes of rTSA after a follow-up of 1 year without revision surgery [36]. These findings support the suggestion to reduce the age limit for rTSA to > 65 years, as performed in our study.

## Limitations

There are several limitations to be considered when interpreting the presented results. At first, the retrospective analysis of the data of our in-house fracture register may be inaccurate and may not provide the quality of a prospective data selection. Second, all patients were queried to assess major complications leading to surgical revision and the electronic medical record was reviewed. Therefore, minor complications such as superficial wound healing problems or numbness could be missed. However, the objective of our study was to assess the major complications leading to surgical revision. At third, there was a significant difference regarding follow-up in both groups. As mentioned above at the beginning of the study in 2007 rTSA presented a low incidence compared to LCP fixation. Our study was based on a self-assessment questionnaire to focus on subjective patient satisfaction. A selection bias regarding patients who did return the questionnaire and those who did not cannot be excluded. Eventually, patients may respond more often to the question which patients were particularly satisfied or dissatisfied. Future investigations with prospective randomized comparisons of LCP fixation and rTSA in PHFs of the elderly are warranted.

## Conclusions

Reversed total shoulder arthroplasty as well as locking plate fixation are both established surgical procedures for the treatment of geriatric four-part fractures of the humeral head leading to similar functional outcome results. However, in mid-term follow-up, the revision rate of the rTSA group was significantly lower compared to the patients treated with locking plate fixation. Therefore, primary rTSA should be considered to treat complex humeral head fracture in geriatric patients with an increased complication risk to avoid revision and repeated anaesthesia.

## References

[1] Court-Brown CM, Garg A, McQueen MM (2001). The epidemiology of proximal humeral fractures. Acta Orthop Scand.

[2] Palvanen M, Kannus P, Niemi S, Parkkari J (2006). Update in the epidemiology of proximal humeral fractures. Clin Orthop Relat Res.

[3] Ensrud KE (2013). Epidemiology of fracture risk with advancing age. J Gerontol A Biol Sci Med Sci.

[4] Neer CS (2002). Four-segment classification of proximal humeral fractures: purpose and reliable use. J Shoulder Elbow Surg.

[5] Bell JE, Leung BC, Spratt KF, Koval KJ, Weinstein JD, Goodman DC, Tosteson AN (2011). Trends and variation in incidence, surgical treatment, and repeat surgery of proximal humeral fractures in the elderly. J Bone Joint Surg Am.

[6] Brorson S, Frich LH, Winther A, Hrobjartsson A (2011). Locking plate osteosynthesis in displaced 4-part fractures of the proximal humerus. Acta Orthop.

[7] Barlow JD, Logli AL, Steinmann SP, Sems SA, Cross WW, Yuan BJ, Torchia ME, Sanchez-Sotelo J (2020). Locking plate fixation of proximal humerus fractures in patients older than 60 years continues to be associated with a high complication rate. J Shoulder Elbow Surg.

[8] Thanasas C, Kontakis G, Angoules A, Limb D, Giannoudis P (2009). Treatment of proximal humerus fractures with locking plates: a systematic review. J Shoulder Elbow Surg.

[9] Evered LA, Silbert BS (2018). Postoperative cognitive dysfunction and noncardiac surgery. Anesth Analg.

[10] Evered L, Scott DA, Silbert B (2017). Cognitive decline associated with anesthesia and surgery in the elderly: does this contribute to dementia prevalence?. Curr Opin Psychiatry.

[11] Seidl A, Sholder D, Warrender W, Livesey M, Williams G, Abboud J, Namdari S (2017). Early versus late reverse shoulder arthroplasty for proximal humerus fractures: does it matter?. Arch Bone Joint Surg.

[12] Sebastia-Forcada E, Lizaur-Utrilla A, Cebrian-Gomez R, Miralles-Munoz FA, Lopez-Prats FA (2017). Outcomes of reverse total shoulder arthroplasty for proximal humeral fractures: primary arthroplasty versus secondary arthroplasty after failed proximal humeral locking plate fixation. J Orthop Trauma.

[13] Neer CS (1970). Displaced proximal humeral fractures. I. Classification and evaluation. J Bone Joint Surg Am.

[14] Schmidutz F, Beirer M, Braunstein V, Bogner V, Wiedemann E, Biberthaler P (2012). The Munich Shoulder Questionnaire (MSQ): development and validation of an effective patient-reported tool for outcome measurement and patient safety in shoulder surgery. Patient Saf Surg.

[15] Beirer M, Crönlein M, Venjakob A, Saier T, Schmitt-Sody M, Huber-Wagner S, Biberthaler P, Kirchhoff C (2015). Additional calcar support using a blade device reduces secondary varus displacement following reconstruction of the proximal humerus: a prospective study. Eur J Med Res.

[16] Greve F, Beirer M, Zyskowski M, Cronlein M, Muller M, Pesch S, Felix S, Biberthaler P, Buchholz A, Kirchhoff C (2019). Prospective outcome analysis following tenodesis of the long head of the biceps tendon along with locking plate osteosynthesis for proximal humerus fractures. Injury.

[17] Capuano L, Poulain S, Hardy P, Longo UG, Denaro V, Maffulli N (2011). No correlation between physicians administered elbow rating systems and patient’s satisfaction. J Sports Med Phys Fitness.

[18] Giardella A, Ascione F, Mocchi M, Berlusconi M, Romano AM, Oliva F, Maradei L (2017). Reverse total shoulder versus angular stable plate treatment for proximal humeral fractures in over 65 years old patients. Muscles Ligaments Tendons J.

[19] Bufquin T, Hersan A, Hubert L, Massin P (2007). Reverse shoulder arthroplasty for the treatment of three- and four-part fractures of the proximal humerus in the elderly: a prospective review of 43 cases with a short-term follow-up. J Bone Joint Surg Br.

[20] Cazeneuve JF, Cristofari DJ (2010). The reverse shoulder prosthesis in the treatment of fractures of the proximal humerus in the elderly. J Bone Joint Surg Br.

[21] Tavakkolizadeh A, Ghassemi A, Colegate-Stone T, Latif A, Sinha J (2009). Gender-specific Constant score correction for age. Knee Surg Sports Traumatol Arthrosc.

[22] Hertel R, Hempfing A, Stiehler M, Leunig M (2004). Predictors of humeral head ischemia after intracapsular fracture of the proximal humerus. J Shoulder Elbow Surg.

[23] Bastian JD, Hertel R (2008). Initial post-fracture humeral head ischemia does not predict development of necrosis. J Shoulder Elbow Surg.

[24] von Ruden C, Augat P (2016). Failure of fracture fixation in osteoporotic bone. Injury.

[25] Augat P, Simon U, Liedert A, Claes L (2005). Mechanics and mechano-biology of fracture healing in normal and osteoporotic bone. Osteoporos Int.

[26] Ackermann O, Stanjek M, Rulander C, von Schulze PC (2012). Proximal cut-out of the hip screw: cement augmentation as a minimally invasive rescue procedure. Zeitschrift fur Orthopadie und Unfallchirurgie.

[27] Katthagen JC, Lutz O, Voigt C, Lill H, Ellwein A (2018). Cement augmentation of humeral head screws reduces early implant-related complications after locked plating of proximal humeral fractures. Obere Extrem.

[28] Nho SJ, Brophy RH, Barker JU, Cornell CN, MacGillivray JD (2007). Management of proximal humeral fractures based on current literature. J Bone Joint Surg Am.

[29] Schafer T, Bohler E, Ruhdorfer S, Weigl L, Wessner D, Filipiak B, Wichmann HE, Ring J (2001). Epidemiology of contact allergy in adults. Allergy.

[30] Akil S, Newman JM, Shah NV, Ahmed N, Deshmukh AJ, Maheshwari AV (2018). Metal hypersensitivity in total hip and knee arthroplasty: current concepts. J Clin Orthop Trauma.

[31] Haasters F, Siebenburger G, Helfen T, Daferner M, Bocker W, Ockert B (2016). Complications of locked plating for proximal humeral fractures—are we getting any better?. J Shoulder Elbow Surg.

[32] Harjula JNE, Paloneva J, Haapakoski J, Kukkonen J, Äärimaa V, Registry FSA, G (2018). Increasing incidence of primary shoulder arthroplasty in Finland—a nationwide registry study. BMC Musculoskelet Disord.

[33] Gupta AK, Harris JD, Erickson BJ, Abrams GD, Bruce B, McCormick F, Nicholson GP, Romeo AA (2015). Surgical management of complex proximal humerus fractures—a systematic review of 92 studies including 4500 patients. J Orthop Trauma.

[34] van Ochten JHM, van der Pluijm M, Pouw M, Felsch QTM, Heesterbeek P, de Vos MJ (2019). Long-term survivorship and clinical and radiological follow-up of the primary uncemented Delta III reverse shoulder prosthesis. J Orthop.

[35] Robinson CM, Stirling PHC, Goudie EB, MacDonald DJ, Strelzow JA (2019). Complications and long-term outcomes of open reduction and plate fixation of proximal humeral fractures. J Bone Joint Surg Am.

[36] Spross C, Meester J, Mazzucchelli RA, Puskas GJ, Zdravkovic V, Jost B (2019). Evidence-based algorithm to treat patients with proximal humerus fractures—a prospective study with early clinical and overall performance results. J Shoulder Elbow Surg.

