# Correction to: Reconstruction or replacement? A challenging question in surgical treatment of complex humeral head fractures in the elderly

**DOI:** 10.1007/s00402-021-04267-3

**Published:** 2021-11-24

**Authors:** M. Müller, F. Greve, M. Crönlein, M. Zyskowski, S. Pesch, P. Biberthaler, C. Kirchhoff, M. Beirer

**Affiliations:** Department of Trauma Surgery, Klinikum rechts der Isar, Technical University Munich, Ismaningerstraße 22, 81675 München, Germany

## Correction to: Archives of Orthopaedic and Trauma Surgery 10.1007/s00402-021-04124-3

The original version of this article unfortunately contained a mistake. There is a mistake in the abstract.

In abstract, second sentence of the result section should read as

63 patients were treated using the LCP fixation compared to 40 rTSAs.

The original article has been corrected.

